# Crystal structures of new psychoactive substances 4-HO-DET and 4-AcO-DET

**DOI:** 10.1107/S2056989026009217

**Published:** 2026-09-08

**Authors:** Marilyn Naeem, Christopher Pauli, Caleb King, Andrew R. Chadeayne, James A. Golen, David R. Manke

**Affiliations:** a University of Massachusetts Dartmouth 285 Old Westport Road North Dartmouth MA 02747 USA; b Tryptomics, LLC, 425 S. Bowen St. Ste 4, Longmont, CO 80501, USA; c CaaMTech, Inc., 58 Sunset Way, Suite 209, Issaquah, WA 98027, USA; Katholieke Universiteit Leuven, Belgium

**Keywords:** crystal structure, tryptamines, indoles, hydrogen bonds

## Abstract

The solid-state structures of 4-hy­droxy-*N*,*N*-di­ethyl­tryptammonium (4-HO-DET) hydro­fumarate {systematic name: dieth­yl[2-(4-hy­droxy-1*H*-indol-3-yl)eth­yl]aza­nium 3-carb­oxy­prop-2-enoate}, C_14_H_21_N_2_O^+^·C_4_H_3_O_4_^−^, and 4-acet­oxy-*N*,*N*-di­ethyl­tryptammonium (4-AcO-DET) fumarate/fumaric acid {systematic name: bis­{[2-(4-acet­oxy-1*H*-indol-3-yl)eth­yl]di­ethyl­aza­nium} but-2-ene­dio­ate–(*E*)-butenedioic acid (1/1)}, 2C_16_H_23_N_2_O^2+^·C_4_H_2_O_4_^2−^·C_4_H_4_O_4_, were determined by single-crystal X-ray diffraction. The 4-HO-DET structure exhibits a single tryptammonium cation and a single hydro­fumarate anion in the asymmetric unit. The ions are joined together by N—H⋯O and O—H⋯O hydrogen bonds in two-dimensional sheets lying in the (001) plane. The 4-AcO-DET structure exhibits a single tryptammonium cation, half of a fumarate dianion and half of a fumaric acid mol­ecule in the asymmetric unit. The ions and mol­ecules are joined together by N—H⋯O and O—H⋯O hydrogen bonds in an infinite three-dimensional network.

## Abstract 2

The solid-state structures of two synthetic psychoactive compounds that have recently appeared in products on the ‘magic mushroom’ retail market are presented.

## 1. Chemical context

Psychedelics, entactogens and psychoplastogens have gained a great deal of inter­est recently in the treatment of major depressive disorder, generalised anxiety disorders, and treatment-resistant depression (Hoyer, 2025 ▸). Psilocybin (4-phosphor­yloxy-*N*,*N*-di­methyl­tryptamine) is a natural product found in ‘magic mushrooms’, and is one traditional psychedelic that is being explored to treat mental health disorders. Psilocybin enzymatically hydrolyses to its active metabolite psilocin (4-hy­droxy-*N*,*N*-di­methyl­tryptamine), which is the primary psychoactive compound found in ‘magic mushrooms’ due to its action as a potent agonist of the serotonin 2A receptor (Glatfelter, Pottie *et al.*, 2022 ▸). In 1959, Albert Hofmann reported the synthesis of the diethyl derivative of psilocybin, ethocybin (4-phosphor­yloxy-*N*,*N*-di­ethyl­tryptamine), and the diethyl derivative of psilocin, ethocin (4-hy­droxy-*N*,*N*-di­ethyl­tryptamine; 4-HO-DET) (Troxler *et al.*, 1959 ▸). In 1989, Jochem Gartz showed that both of these diethyl derivatives can be produced in mushrooms by introducing the unsubstituted tryptamine, *N*,*N*-di­ethyl­tryptamine, to the mycelium of *Psilocybe* mushrooms (Gartz, 1989 ▸). 4-HO-DET shows strong binding at the serotonin 2A receptor and is known to be psychoactive. There is also a synthetic prodrug of 4-HO-DET, 4-acet­oxy-*N*,*N*-di­ethyl­tryptamine (4-AcO-DET), which was first synthesized by Albert Hofmann (Hofmann & Troxler, 1963 ▸). As a prodrug, 4-AcO-DET does not show binding at the serotonin 2A receptor (Glatfelter, Naeem *et al.*, 2023 ▸), but it demonstrates similar molar efficacy to 4-HO-DET in animal studies as it quickly hydrolyses to produce 4-HO-DET (Klein *et al.*, 2021 ▸). Recently, 4-AcO-DET and 4-HO-DET have appeared in products for sale in US retail stores and online. Products marketed as psilocybin-containing ‘magic mushroom’ edibles frequently contain no psilocybin. In a recent analysis of twelve gummy and chocolate products sold as ‘magic mushroom’ edibles, psilocybin was not detected in any sample; the active constituents identified instead included synthetic tryptamines and other undisclosed compounds (van Breeman *et al.*, 2025 ▸).
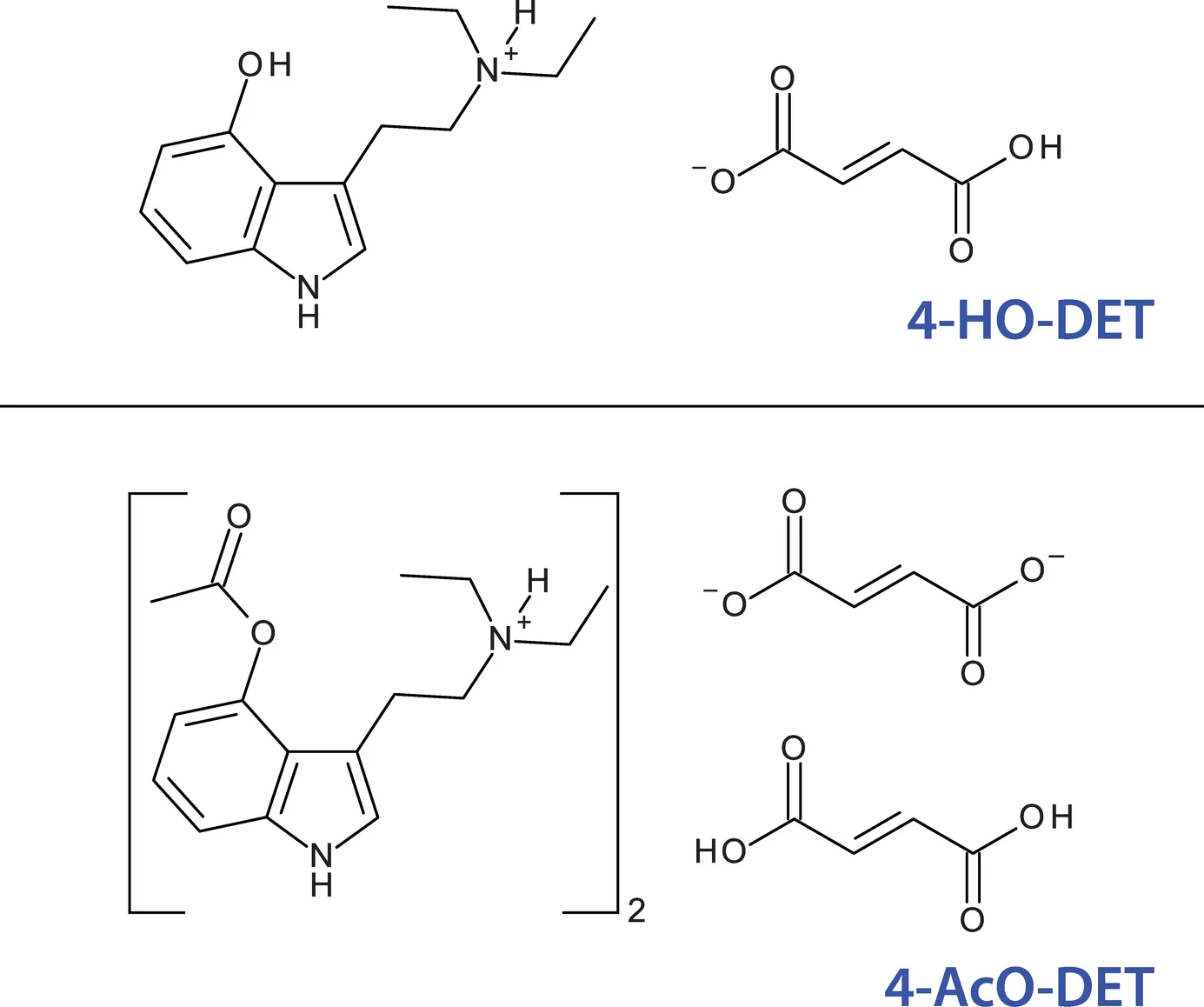


Tryptomics, LLC analyzes commercial samples with a tryptamine assay that provides testing for an array of compounds often found in psychoactive substances. Across anonymously analyzed samples using HPLC-DAD technology following solvent and sonication extraction protocols 4-AcO-DET and/or 4-HO-DET were found in 87 samples. Twenty-five samples that were extracts and isolates showed the widest and highest range of 4-AcO-DET content (2.1–733.5 mg g^−1^), with 4-HO-DET content reaching a maximum value of 981.8 mg g^−1^. Sixty samples of infused edibles had maximum concentrations of 16.42 and 37.02 mg g^−1^ for 4-AcO-DET and 4-HO-DET, respectively; these concentrations corresponded to edible package contents of 58.8 mg/package for 4-AcO-DET and 72.9 mg/package for 4-HH-DET. One inhalable sample contained 14.6 mg g^−1^ of 4-AcO-DET. Reported orally actived doses of 4-AcO-DET are on the order of tens of milligrams (Shulgin & Shulgin, 1997 ▸). The per-package contents measured here therefore correspond to several such doses in a single retail unit. As these new psychoactive substances become more prevalent in the marketplace, the study of these materials and their properties becomes more important. As such the crystal structures of 4-hy­droxy-*N*,*N*-di­ethyl­tryptamine as its hydro­fumarate salt and 4-acet­oxy-*N*,*N*-di­ethyl­tryptamine as a fumarate salt with a fumaric acid adduct are reported.

## 2. Structural commentary

**Figure 1:**
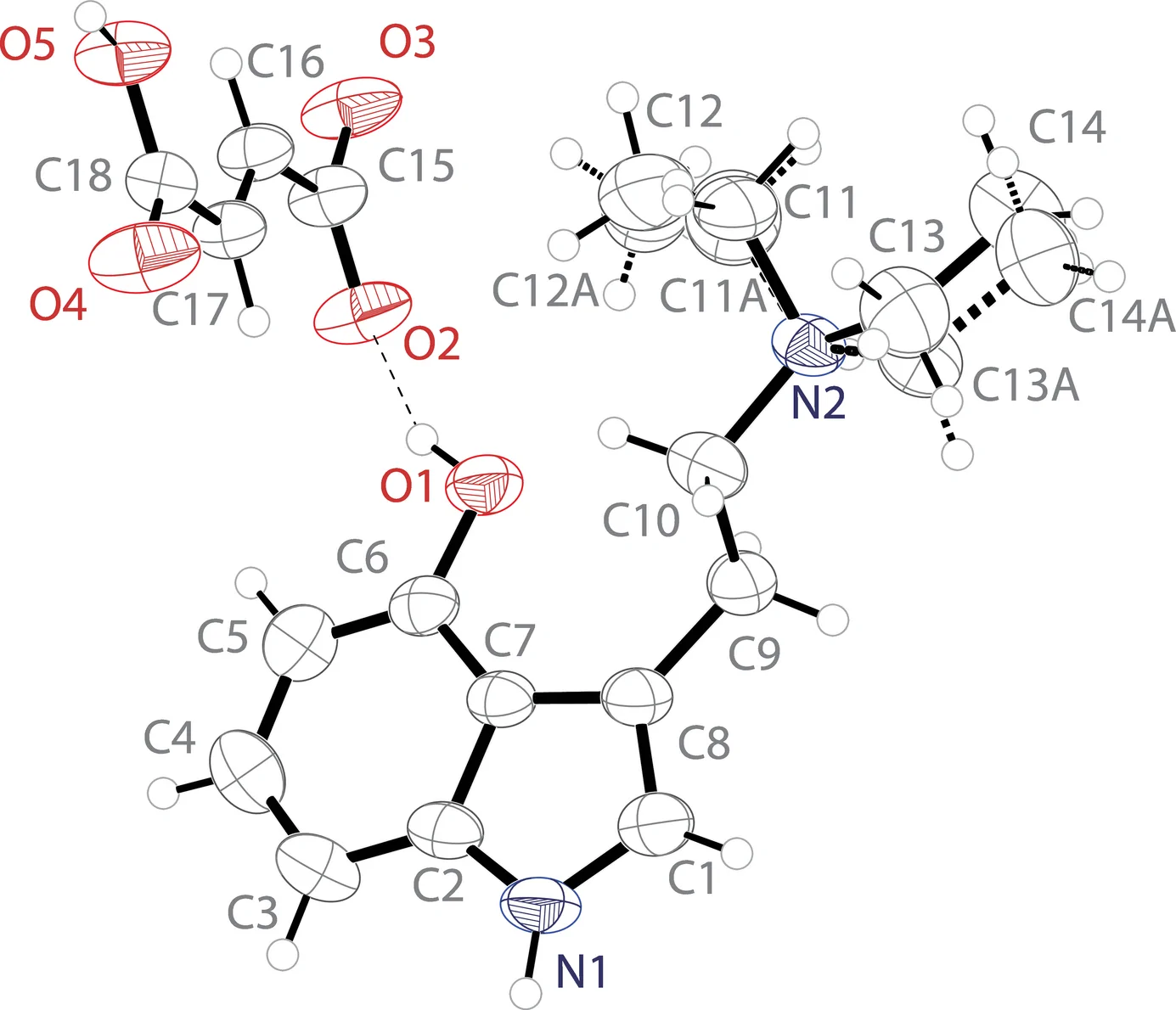
The mol­ecular structure of 4-HO-DET hydro­fumarate showing the atomic labeling. Displacement ellipsoids are drawn at the 50% probability level. Dashed bonds indicate a disordered component in the structure. Hydrogen bonds are shown as dashed lines.

The mol­ecular structure of 4-HO-DET hydro­fumarate is shown in Fig. 1 ▸. The asymmetric unit contains one 4-hy­droxy-*N*,*N*-di­ethyl­tryptammonium (C_14_H_21_N_2_O^+^) cation and one hydro­fumarate (C_4_H_3_O_4_^−^) anion. The indole ring of the cation is near planar with a r.m.s. deviation from planarity of 0.007 Å for the non-hydrogen atoms. The hydro­fumarate anion is slightly less planar with a r.m.s. deviation from planarity of 0.053 Å for non-hydrogen atoms. The ethyl­amino arm of the tryptamine is turned away from the indole plane with a C7—C8—C9—C10 torsion angle of 76.0 (3)° and the C8—C9—C10—N2 grouping has an *anti* conformation [torsion angle = 177.2 (2)°]. Each ethyl group is disordered over two orientations with C11 and C12 showing a 0.80 (3):0.20 (3) ratio and C13 and C14 showing a 0.82 (2):0.18 (2) ratio.

**Figure 2:**
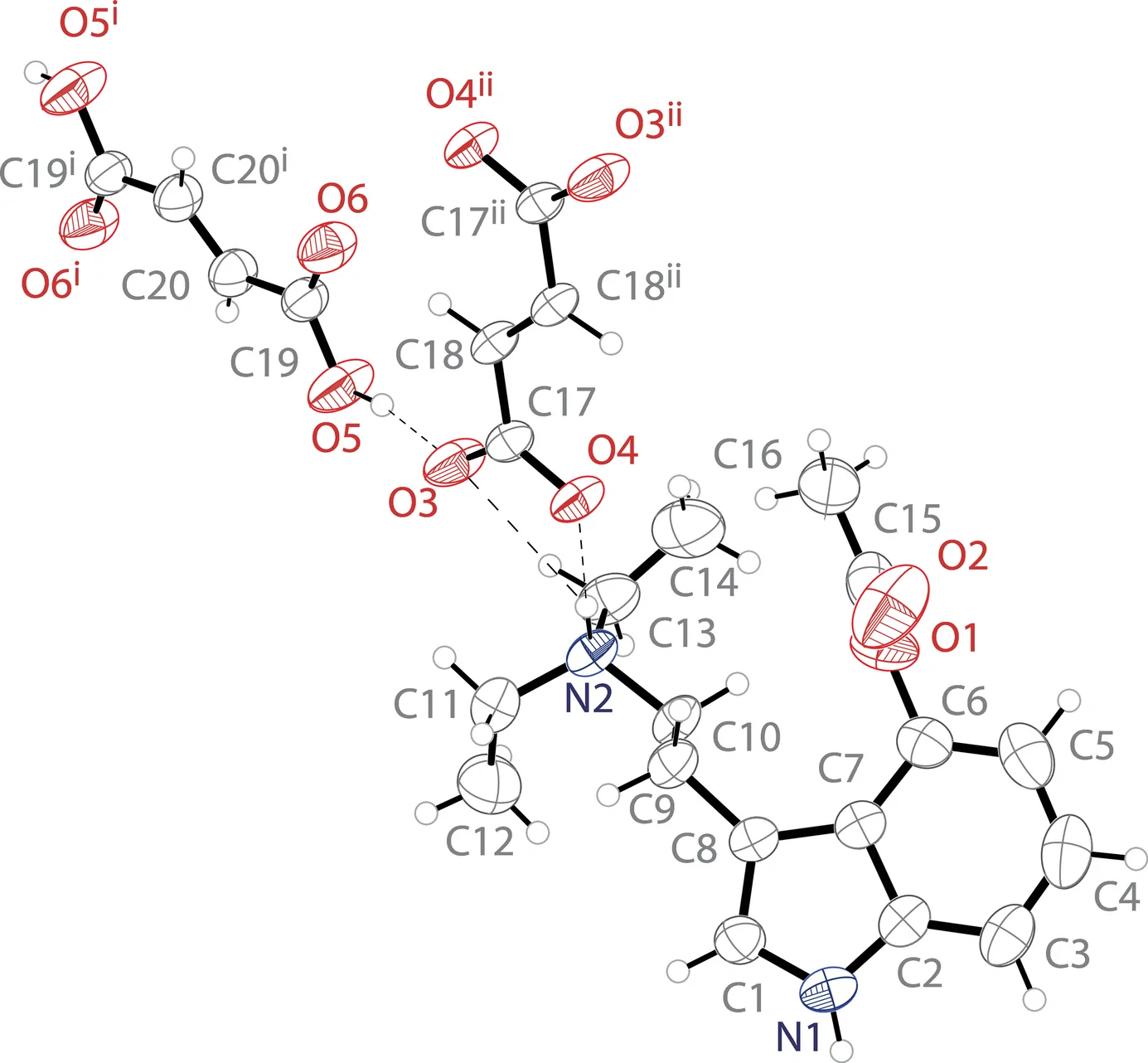
The mol­ecular structure of 4-AcO-DET fumarate/fumaric acid showing the atomic labeling. Displacement ellipsoids are drawn at the 50% probability level. Hydrogen bonds are shown as dashed lines. Symmetry codes: (i) 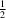 − *x*, 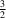 − *y*, 1 − *z*; (ii) 1 − *x*, 2 − *y*, 1 − *z*.

The mol­ecular structure of 4-AcO-DET fumarate/fumaric acid is shown in Fig. 2 ▸. The asymmetric unit contains one 4-acet­oxy-*N*,*N*-di­ethyl­tryptammonium (C_16_H_23_N_2_O_2_^+^) cation, one half of a fumarate (C_4_H_2_O_4_^2−^) dianion, and one half of a fumaric acid (C_4_H_4_O_4_) mol­ecule. The indole ring system of the cation is near planar with a r.m.s. deviation from planarity of 0.013 Å. The ethyl­amino arm is turned away from the indole plane with a C7—C8—C9—C10 torsion angle of 73.5 (3)° and the C8—C9—C10—N2 grouping has an *anti* conformation [torsion angle = 174.0 (2)°]. The complete fumarate dianion is generated through inversion, and is near planar with a r.m.s. deviation from planarity of 0.025 Å. The complete fumaric acid dianion is generated similarly and only slightly less planar with a r.m.s. deviation of 0.044 Å.

## 3. Supra­molecular features

**Figure 3:**
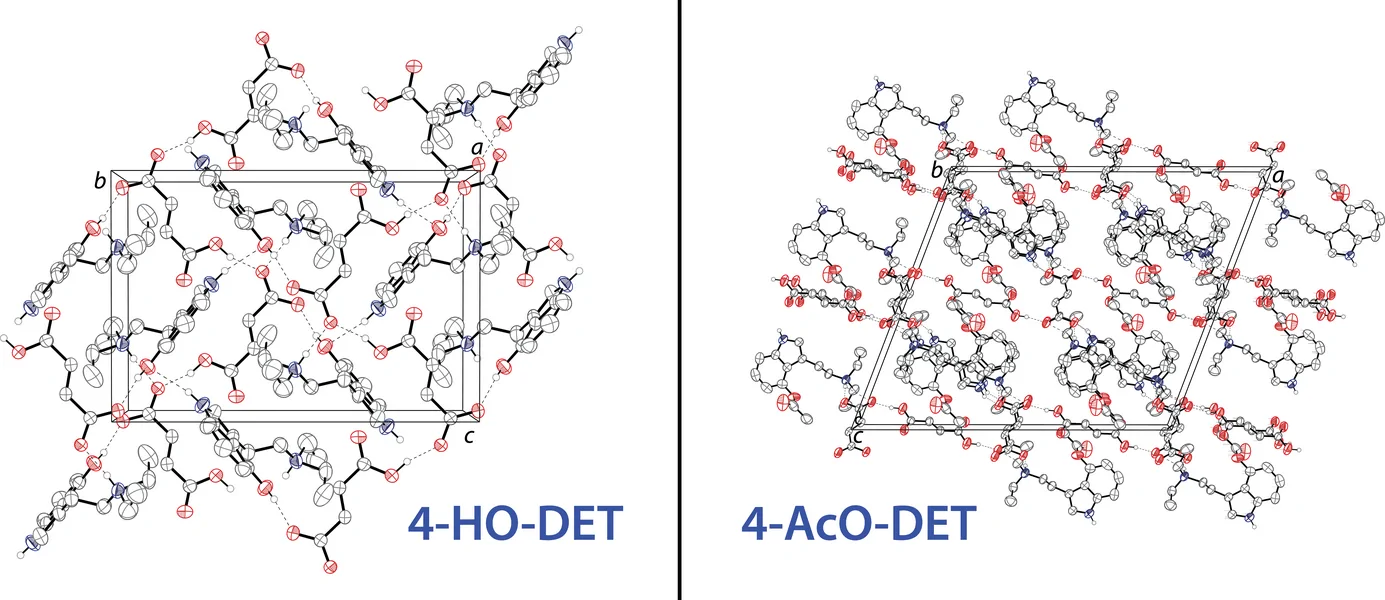
The crystal packing of 4-HO-DET hydro­fumarate (left) viewed along the *a-*axis direction, and the crystal packing of 4-AcO-DET fumarate/fumaric acid (right) views along the *a*-axis direction. The hydrogen bonds (Tables 1 ▸ and 2 ▸) are shown as dashed lines. Displacement ellipsoids are drawn at the 50% probability level. Hydrogen atoms not involved in hydrogen bonding are omitted for clarity. Only the major components of disordered residues are shown.

In 4-HO-DET hydro­fumarate, the hy­droxy groups of the tryptammonium cations are connected to the hydro­fumarate anion in the asymmetric unit via O1—H1⋯O2 hydrogen bonds. The carb­oxy­lic acid groups of the hydro­fumarate anions are connected to another hydro­fumarate anion via O5—H5*A*··O3 hydrogen bonds. The indole nitro­gen atoms are connected with the hy­droxy group of another tryptammonium cation through N1—H1⋯O1 hydrogen bonds. Finally, the ethyl­ammonium nitro­gen hydrogen bonds through N2—H2⋯O3 inter­actions with hydro­fumarate anions. These hydrogen bonds are summarized in Table 1 ▸, and join the ions in the solid together into two-dimensional sheets lying in the (100) plane. The packing of these ions is shown in Fig. 3 ▸ (left).

In 4-AcO-DET fumarate/fumaric acid, the ethyl­ammonium nitro­gen has a bifurcated hydrogen bond with the two oxygen atoms of the fumaric acid as N2—H2⋯(O3,O4) inter­actions. The indole N atom hydrogen bonds to one of the O atoms of the fumaric acid via N1—H1⋯O4 inter­actions. The fumaric acid also hydrogen bonds to the fumarate dianion through the O5—H5*A*⋯O3 inter­action. These hydrogen bonds are summarized in Table 2 ▸, and join the ions in the solid together into an infinite three-dimensional network. The packing of these ions is shown in Fig. 3 ▸ (right).

## 4. Database survey

There are 14 crystal structures of 4-hy­droxy­tryptamines reported in the literature. These are the natural products psilocin, 4-hy­droxy-*N*,*N*-di­methyl­tryptamine, as its freebase (Petcher & Weber, 1974 ▸; CCDC refcode PSILIN) and norpsilocin, 4-hy­droxy-*N*-methyl­tryptamine, as its freebase and fumarate salt (Chadeayne *et al.*, 2020*c* ▸; MULXAV, MULXEZ). Three synthetic variants of norpsilocin have been reported, 4-hy­droxy-*N*-iso­propyl­tryptamine (Laban *et al.*, 2023 ▸; YEYZOV), 4-hy­droxy-*N*-*n*-propyl­tryptamine and 4-hy­droxy-*N*-ethyl­tryptamine (Naeem *et al.*, 2026 ▸). The synthetic variants of psilocin that have been reported are 4-hy­droxy-*N*,*N*-diiso­propyl­tryptamine as its hydro­fumarate salt (Naeem *et al.*, 2025 ▸; BUWSOF), 4-hy­droxy-*N*,*N*-di-*n*-propyl­tryptamine as its chloride (Sammeta *et al.*, 2020 ▸; WAMGEA) and fumarate (Chadeayne, Pham *et al.*, 2019*b* ▸; WUCGAF) salts, and 4-hy­droxy-*N*-methyl-*N*-iso­propyl­tryptamine as its hydro­fumarate (Chadeayne, Pham *et al.*, 2019*a* ▸; RONSUL) and fumarate (Chadeayne, 2020*b* ▸; TUFQAP) salts. There are also four quaternary 4-hy­droxy­tryptamines that are reported as their iodide salts in 4-hy­droxy-*N*,*N*,*N*-tri­methyl­tryptammonium (Chadeayne *et al.*, 2020*a* ▸; XUXFAA), 4-hy­droxy-*N*,*N*-dimethyl-*N*-ethyl­tryptammonium, 4-hy­droxy-*N*,*N*-dimethyl-*N*-*n*-propyl­tryptammonium and 4-hy­droxy-*N*,*N*-dimethyl-*N*-iso­propyl­tryptammonium (Glatfelter, Pham *et al.*, 2022 ▸; EDOYIJ, EDOYUV, EDOZIK).

There are 11 crystal structures of 4-acet­oxy­tryptamines reported in the literature. There is one monoalkyltryptamine with 4-acet­oxy-*N*-methyl­tryptamine (Glatfelter, Pottie *et al.*, 2022 ▸). There are four quaternary 4-acet­oxy­tryptamines reported as their iodide salts in 4-acet­oxy-*N*,*N*,*N*-tri­methyl­tryptammonium (Chadeayne *et al.*, 2020*a* ▸; XUXDUS), 4-acet­oxy-*N*,*N*-dimethyl-*N*-ethyl­tryptammonium, 4-acet­oxy-*N*,*N*-dimethyl-*N*-*n*-propyl­tryptammonium and 4-acet­oxy-*N*,*N*-dimethyl-*N*-iso­propyl­tryptammonium (Glatfelter, Pham *et al.*, 2022 ▸; EDOZEG, EDOYOP, EDOZAC). There are six di­alkyl­tryptamines with 4-acet­oxy-*N*-ethyl-*N*-*n*-propyl­trypt­amine as a fumarate/fumaric acid compound (Pham *et al.*, 2023 ▸; BIYKED), 4-acet­oxy-*N*-methyl-*N*-ethyl­tryptamine as its hydro­fumarate salt, 4-acet­oxy-*N*-methyl-*N*-allyl­tryptamine as its hydro­fumarate salt, 4-acet­oxy-*N*,*N*-di­allyl­tryptamine as a fumarate/fumaric acid compound (Pham *et al.*, 2021 ▸; OJIQIK, OJIQOQ, OJIQUW), and 4-acet­oxy-*N*,*N*-di­methyl­tryptamine as both fumarate (Chadeayne, Golen *et al.*, 2019*b* ▸; XOFDOO) and hydro­fumarate (Chadeayne, Golen *et al.*, 2019*a* ▸; HOCJUH) salts. The structure of 4-acet­oxy-*N*,*N*-di­allyl­tryptammonium fumarate/fumaric acid is the most similar to the reported structure of 4-acet­oxy-*N*,*N*-di­ethyl­tryptammonium fumarate/fumaric acid, demonstrating the same space group, general packing, and similar unit-cell parameters.

## 5. Synthesis and crystallization

Single crystals of 4-hy­droxy-*N*,*N*-di­ethyl­tryptammonium hydro­fumarate suitable for X-ray analysis were obtained from the slow evaporation of an ethano­lic solution of a commercial sample (ChemLogix). Single crystals of 4-acet­oxy-*N*,*N*-di­ethyl­tryptammonium fumarate/fumaric acid suitable for X-ray analysis were grown from the slow evaporation of a methanol/acetone solution of a commercial sample (The Indole Shop).

## 6. Refinement

Crystal data, data collection and structure refinement details are summarized in Table 3 ▸. The H atoms bonded to C atoms were included in the refinement using riding models. The H atoms bonded to heteroatoms (N and O) were located in difference-Fourier maps and were refined isotropically with the use of DFIX commands. The ethyl groups in 4-HO-DET are disordered over two orientations and refined with the use of DFIX, SADI and DELU commands resulting in occupancies for C11 and C12 of 0.80 (3):0.20 (3), and for C13 and C14 of 0.82 (2):0.18 (2).

## Supplementary Material

Crystal structure: contains datablock(s) 4-HO-DET, 4-AcO-DET. DOI: 10.1107/S2056989026009217/vm2336sup1.cif

Structure factors: contains datablock(s) 4-HO-DET. DOI: 10.1107/S2056989026009217/vm23364-HO-DETsup2.hkl

Structure factors: contains datablock(s) 4-AcO-DET. DOI: 10.1107/S2056989026009217/vm23364-AcO-DETsup3.hkl

Supporting information file. DOI: 10.1107/S2056989026009217/vm23364-HO-DETsup4.cml

Supporting information file. DOI: 10.1107/S2056989026009217/vm23364-AcO-DETsup5.cml

CCDC references: 2585205, 2585204

Additional supporting information:  crystallographic information; 3D view; checkCIF report

## Figures and Tables

**Table 1:** Hydrogen-bond geometry (Å, °) for 4-HO-DET[Chem scheme1]

*D*—H⋯*A*	*D*—H	H⋯*A*	*D*⋯*A*	*D*—H⋯*A*
O5—H5*A*⋯O3^i^	0.90 (1)	1.70 (2)	2.559 (2)	157 (4)
O1—H1⋯O2	0.87 (1)	1.79 (2)	2.607 (3)	155 (4)
N1—H1*A*⋯O1^ii^	0.87 (1)	2.15 (1)	3.011 (3)	169 (3)
N2—H2⋯O3^iii^	0.85 (1)	1.96 (1)	2.783 (3)	165 (3)

Symmetry codes: (i) 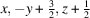; (ii) 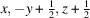; (iii) 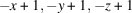.

**Table 2:** Hydrogen-bond geometry (Å, °) for 4-AcO-DET[Chem scheme1]

*D*—H⋯*A*	*D*—H	H⋯*A*	*D*⋯*A*	*D*—H⋯*A*
N1—H1⋯O4^i^	0.87 (1)	2.12 (2)	2.876 (3)	145 (3)
N2—H2⋯O3	0.91 (1)	2.65 (2)	3.241 (2)	124 (2)
N2—H2⋯O4	0.91 (1)	1.91 (1)	2.818 (2)	177 (3)
O5—H5*A*⋯O3	0.95 (1)	1.62 (1)	2.558 (2)	167 (3)

Symmetry code: (i) 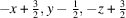.

**Table 3:** Experimental details

	4-HO-DET	4-AcO-DET
Crystal data		
Chemical formula	C_14_H_21_N_2_O^+^·C_4_H_3_O_4_^−^	2(C_16_H_23_N_2_O_2_^+^)·C_4_H_2_O_4_^2−^·C_4_H_4_O_4_
*M*_r_	348.39	390.43
Crystal system, space group	Monoclinic, *P*2_1_/*c*	Monoclinic, *C*2/*c*
Temperature (K)	300	300
*a*, *b*, *c* (Å)	10.9690 (9), 15.6452 (12), 10.6839 (7)	24.410 (2), 8.4508 (7), 21.445 (2)
β (°)	92.084 (2)	110.963 (3)
*V* (Å^3^)	1832.3 (2)	4131.1 (7)
*Z*	4	8
Radiation type	Mo *K*α	Mo *K*α
μ (mm^−1^)	0.09	0.09
Crystal size (mm)	0.42 × 0.3 × 0.12	0.18 × 0.15 × 0.12

Data collection		
Diffractometer	Bruker D8 Venture CMOS	Bruker D8 Venture CMOS
Absorption correction	Multi-scan (*SADABS*; Krause *et al.*, 2015 ▸)	Multi-scan (*SADABS*; Krause *et al.*, 2015 ▸)
*T*_min_, *T*_max_	0.580, 0.745	0.697, 0.745
No. of measured, independent and observed [*I* > 2σ(*I*)] reflections	46191, 3499, 2735	47661, 3525, 2420
*R*_int_	0.062	0.113
(sin θ/λ)_max_ (Å^−1^)	0.612	0.588

Refinement		
*R*[*F*^2^ > 2σ(*F*^2^)], *wR*(*F*^2^), *S*	0.056, 0.164, 1.10	0.048, 0.122, 1.04
No. of reflections	3499	3525
No. of parameters	284	268
No. of restraints	67	9
H-atom treatment	H atoms treated by a mixture of independent and constrained refinement	H atoms treated by a mixture of independent and constrained refinement
Δρ_max_, Δρ_min_ (e Å^−3^)	0.34, −0.19	0.33, −0.17

Computer programs: *APEX4* and *SAINT* (Bruker, 2025 ▸), SHELXT2014 (Sheldrick, 2015*a* ▸), *SHELXL2018* (Sheldrick, 2015*b* ▸), *OLEX2* (Dolomanov *et al.*, 2009 ▸) and *publCIF* (Westrip, 2010 ▸).

## supplementary crystallographic information

### Diethyl[2-(4-hydroxy-1H-indol-3-yl)ethyl]azanium 3-carboxyprop-2-enoate (4-HO-DET) Crystal data

**Table:**

C_14_H_21_N_2_O^+^·C_4_H_3_O_4_^−^	*F*(000) = 744
*M**_r_* = 348.39	*D*_x_ = 1.263 Mg m^−^^3^
Monoclinic, *P*2_1_/*c*	Mo *K*α radiation, λ = 0.71073 Å
*a* = 10.9690 (9) Å	Cell parameters from 9923 reflections
*b* = 15.6452 (12) Å	θ = 2.6–25.7°
*c* = 10.6839 (7) Å	µ = 0.09 mm^−^^1^
β = 92.084 (2)°	*T* = 300 K
*V* = 1832.3 (2) Å^3^	BLOCK, colourless
*Z* = 4	0.42 × 0.3 × 0.12 mm

### Diethyl[2-(4-hydroxy-1H-indol-3-yl)ethyl]azanium 3-carboxyprop-2-enoate (4-HO-DET) Data collection

**Table:**

Bruker D8 Venture CMOS diffractometer	2735 reflections with *I* > 2σ(*I*)
φ and ω scans	*R*_int_ = 0.062
Absorption correction: multi-scan (SADABS; Krause *et al.*, 2015)	θ_max_ = 25.8°, θ_min_ = 2.6°
*T*_min_ = 0.580, *T*_max_ = 0.745	*h* = −13→13
46191 measured reflections	*k* = −19→19
3499 independent reflections	*l* = −13→12

### Diethyl[2-(4-hydroxy-1H-indol-3-yl)ethyl]azanium 3-carboxyprop-2-enoate (4-HO-DET) Refinement

**Table:**

Refinement on *F*^2^	Primary atom site location: structure-invariant direct methods
Least-squares matrix: full	Hydrogen site location: mixed
*R*[*F*^2^ > 2σ(*F*^2^)] = 0.056	H atoms treated by a mixture of independent and constrained refinement
*wR*(*F*^2^) = 0.164	*w* = 1/[σ^2^(*F*_o_^2^) + (0.0498*P*)^2^ + 1.6679*P*] where *P* = (*F*_o_^2^ + 2*F*_c_^2^)/3
*S* = 1.10	(Δ/σ)_max_ < 0.001
3499 reflections	Δρ_max_ = 0.34 e Å^−^^3^
284 parameters	Δρ_min_ = −0.19 e Å^−^^3^
67 restraints	

### Diethyl[2-(4-hydroxy-1H-indol-3-yl)ethyl]azanium 3-carboxyprop-2-enoate (4-HO-DET) Special details

**Table:**

Geometry. All esds (except the esd in the dihedral angle between two l.s. planes) are estimated using the full covariance matrix. The cell esds are taken into account individually in the estimation of esds in distances, angles and torsion angles; correlations between esds in cell parameters are only used when they are defined by crystal symmetry. An approximate (isotropic) treatment of cell esds is used for estimating esds involving l.s. planes.

### Diethyl[2-(4-hydroxy-1H-indol-3-yl)ethyl]azanium 3-carboxyprop-2-enoate (4-HO-DET) Fractional atomic coordinates and isotropic or equivalent isotropic displacement parameters (Å^2^)

**Table:**

	*x*	*y*	*z*	*U*_iso_*/*U*_eq_	Occ. (<1)
O1	0.34358 (19)	0.41479 (12)	0.71108 (17)	0.0522 (5)	
O2	0.2169 (2)	0.49336 (11)	0.53482 (17)	0.0599 (6)	
O3	0.2145 (2)	0.59686 (11)	0.39464 (16)	0.0629 (6)	
O4	0.1874 (2)	0.67378 (13)	0.93001 (17)	0.0713 (7)	
O5	0.1643 (2)	0.76553 (11)	0.77401 (17)	0.0558 (5)	
N1	0.4000 (2)	0.23712 (15)	1.0502 (2)	0.0520 (6)	
N2	0.6971 (2)	0.50168 (13)	0.7990 (2)	0.0460 (5)	
C1	0.5095 (3)	0.26678 (17)	1.0111 (3)	0.0541 (7)	
H1B	0.584988	0.252123	1.047254	0.065*	
C2	0.3081 (2)	0.27094 (15)	0.9750 (2)	0.0435 (6)	
C3	0.1832 (3)	0.2587 (2)	0.9772 (3)	0.0592 (8)	
H3	0.148887	0.222588	1.035325	0.071*	
C4	0.1123 (3)	0.3021 (2)	0.8902 (3)	0.0630 (8)	
H4	0.027991	0.295863	0.890565	0.076*	
C5	0.1633 (3)	0.35544 (18)	0.8008 (3)	0.0520 (7)	
H5	0.112732	0.383251	0.742156	0.062*	
C6	0.2868 (2)	0.36705 (15)	0.7990 (2)	0.0410 (6)	
C7	0.3631 (2)	0.32505 (14)	0.8882 (2)	0.0392 (5)	
C8	0.4928 (2)	0.32060 (15)	0.9123 (2)	0.0448 (6)	
C9	0.5922 (2)	0.36298 (16)	0.8423 (3)	0.0489 (6)	
H9A	0.669315	0.335311	0.863418	0.059*	
H9B	0.575828	0.356207	0.752999	0.059*	
C10	0.6016 (3)	0.45621 (17)	0.8730 (3)	0.0530 (7)	
H10A	0.621549	0.462623	0.961710	0.064*	
H10B	0.523007	0.483028	0.855991	0.064*	
C11	0.6538 (5)	0.5871 (3)	0.7564 (8)	0.0597 (17)	0.80 (3)
H11A	0.628697	0.619802	0.828136	0.072*	0.80 (3)
H11B	0.720520	0.617411	0.719005	0.072*	0.80 (3)
C12	0.5519 (7)	0.5809 (6)	0.6654 (11)	0.091 (2)	0.80 (3)
H12A	0.542057	0.634329	0.622087	0.136*	0.80 (3)
H12B	0.478657	0.567686	0.707897	0.136*	0.80 (3)
H12C	0.567983	0.536465	0.606158	0.136*	0.80 (3)
C11A	0.638 (3)	0.569 (2)	0.717 (4)	0.090 (9)	0.20 (3)
H11C	0.614850	0.614272	0.773652	0.108*	0.20 (3)
H11D	0.703801	0.592688	0.668931	0.108*	0.20 (3)
C12A	0.537 (3)	0.561 (3)	0.629 (3)	0.082 (8)	0.20 (3)
H12D	0.482185	0.608488	0.638077	0.124*	0.20 (3)
H12E	0.494331	0.508715	0.643874	0.124*	0.20 (3)
H12F	0.566036	0.560781	0.545039	0.124*	0.20 (3)
C13	0.8072 (16)	0.5348 (14)	0.869 (2)	0.078 (9)	0.18 (2)
H13A	0.782496	0.582344	0.920944	0.093*	0.18 (2)
H13B	0.837871	0.490228	0.925030	0.093*	0.18 (2)
C14	0.906 (4)	0.563 (3)	0.793 (4)	0.088 (10)	0.18 (2)
H14A	0.977771	0.571574	0.845499	0.133*	0.18 (2)
H14B	0.883418	0.615919	0.752859	0.133*	0.18 (2)
H14C	0.921327	0.520605	0.731116	0.133*	0.18 (2)
C13A	0.8131 (4)	0.5074 (5)	0.8827 (5)	0.0603 (16)	0.82 (2)
H13C	0.797340	0.544268	0.953288	0.072*	0.82 (2)
H13D	0.832192	0.450964	0.915395	0.072*	0.82 (2)
C14A	0.9196 (6)	0.5403 (6)	0.8187 (8)	0.081 (2)	0.82 (2)
H14D	0.990161	0.537559	0.874354	0.121*	0.82 (2)
H14E	0.905105	0.598504	0.794044	0.121*	0.82 (2)
H14F	0.933153	0.506213	0.745725	0.121*	0.82 (2)
C15	0.2073 (3)	0.56983 (15)	0.5051 (2)	0.0451 (6)	
C16	0.1899 (2)	0.63673 (15)	0.6032 (2)	0.0433 (6)	
H16	0.178566	0.692838	0.576447	0.052*	
C17	0.1895 (2)	0.62200 (15)	0.7229 (2)	0.0416 (6)	
H17	0.195706	0.565513	0.749521	0.050*	
C18	0.1799 (2)	0.68909 (15)	0.8200 (2)	0.0404 (5)	
H5A	0.169 (4)	0.8081 (18)	0.831 (3)	0.099 (13)*	
H1	0.285 (3)	0.440 (2)	0.668 (3)	0.097 (13)*	
H1A	0.383 (3)	0.1985 (18)	1.105 (3)	0.087 (12)*	
H2	0.711 (2)	0.4712 (15)	0.7356 (17)	0.043 (7)*	

### Diethyl[2-(4-hydroxy-1H-indol-3-yl)ethyl]azanium 3-carboxyprop-2-enoate (4-HO-DET) Atomic displacement parameters (Å^2^)

**Table:**

	*U*^11^	*U*^22^	*U*^33^	*U*^12^	*U*^13^	*U*^23^
O1	0.0642 (12)	0.0451 (10)	0.0478 (11)	0.0062 (9)	0.0079 (9)	0.0135 (8)
O2	0.1038 (17)	0.0329 (9)	0.0428 (10)	0.0092 (10)	−0.0004 (10)	0.0024 (8)
O3	0.1209 (18)	0.0350 (9)	0.0334 (10)	0.0147 (10)	0.0104 (10)	0.0021 (7)
O4	0.129 (2)	0.0509 (12)	0.0333 (10)	0.0010 (12)	−0.0030 (11)	0.0006 (9)
O5	0.0945 (15)	0.0362 (10)	0.0365 (10)	−0.0031 (9)	−0.0003 (9)	−0.0035 (8)
N1	0.0622 (15)	0.0440 (12)	0.0499 (13)	−0.0045 (11)	0.0044 (11)	0.0164 (10)
N2	0.0553 (13)	0.0334 (11)	0.0499 (13)	−0.0082 (9)	0.0105 (10)	−0.0050 (9)
C1	0.0541 (16)	0.0452 (15)	0.0628 (17)	−0.0008 (12)	−0.0002 (13)	0.0129 (13)
C2	0.0564 (15)	0.0324 (12)	0.0421 (13)	−0.0079 (11)	0.0062 (11)	0.0001 (10)
C3	0.0611 (18)	0.0577 (17)	0.0592 (18)	−0.0188 (14)	0.0079 (14)	0.0085 (14)
C4	0.0487 (16)	0.072 (2)	0.0685 (19)	−0.0131 (15)	0.0061 (14)	−0.0015 (16)
C5	0.0536 (16)	0.0541 (16)	0.0479 (15)	0.0024 (13)	−0.0039 (12)	−0.0036 (12)
C6	0.0554 (15)	0.0317 (12)	0.0363 (12)	0.0039 (10)	0.0080 (10)	−0.0021 (9)
C7	0.0507 (14)	0.0288 (11)	0.0385 (12)	−0.0011 (10)	0.0070 (10)	−0.0010 (9)
C8	0.0505 (15)	0.0332 (12)	0.0511 (14)	−0.0023 (10)	0.0064 (11)	0.0038 (10)
C9	0.0517 (15)	0.0384 (13)	0.0573 (16)	−0.0002 (11)	0.0105 (12)	0.0008 (11)
C10	0.0661 (18)	0.0445 (15)	0.0493 (15)	−0.0094 (13)	0.0152 (13)	−0.0037 (12)
C11	0.070 (3)	0.035 (2)	0.074 (3)	−0.0067 (16)	0.009 (2)	0.0007 (18)
C12	0.080 (4)	0.082 (5)	0.110 (5)	−0.002 (3)	−0.010 (3)	0.039 (4)
C11A	0.089 (9)	0.091 (9)	0.090 (9)	0.001 (4)	0.005 (4)	0.003 (4)
C12A	0.079 (10)	0.084 (11)	0.084 (10)	0.005 (7)	0.003 (6)	0.002 (7)
C13	0.077 (9)	0.078 (9)	0.079 (9)	−0.006 (4)	0.007 (4)	−0.007 (4)
C14	0.086 (12)	0.090 (13)	0.090 (12)	−0.014 (7)	0.014 (7)	0.008 (8)
C13A	0.065 (2)	0.062 (3)	0.054 (2)	−0.0123 (18)	0.0010 (16)	−0.003 (2)
C14A	0.061 (3)	0.092 (5)	0.089 (4)	−0.010 (3)	0.003 (3)	−0.004 (3)
C15	0.0656 (17)	0.0348 (12)	0.0349 (13)	0.0034 (11)	0.0009 (11)	0.0005 (10)
C16	0.0611 (16)	0.0299 (12)	0.0390 (13)	0.0014 (11)	0.0032 (11)	0.0002 (10)
C17	0.0541 (15)	0.0314 (12)	0.0394 (13)	−0.0010 (10)	0.0017 (10)	0.0009 (10)
C18	0.0478 (14)	0.0387 (13)	0.0347 (13)	−0.0052 (10)	0.0001 (10)	−0.0008 (10)

### Diethyl[2-(4-hydroxy-1H-indol-3-yl)ethyl]azanium 3-carboxyprop-2-enoate (4-HO-DET) Geometric parameters (Å, º)

**Table:**

O1—C6	1.368 (3)	C10—H10A	0.9700
O1—H1	0.872 (10)	C10—H10B	0.9700
O2—C15	1.241 (3)	C11—H11A	0.9700
O3—C15	1.259 (3)	C11—H11B	0.9700
O4—C18	1.200 (3)	C11—C12	1.458 (7)
O5—C18	1.302 (3)	C12—H12A	0.9600
O5—H5A	0.901 (10)	C12—H12B	0.9600
N1—C1	1.367 (4)	C12—H12C	0.9600
N1—C2	1.372 (3)	C11A—H11C	0.9700
N1—H1A	0.870 (10)	C11A—H11D	0.9700
N2—C10	1.512 (3)	C11A—C12A	1.436 (16)
N2—C11	1.484 (4)	C12A—H12D	0.9600
N2—C11A	1.506 (10)	C12A—H12E	0.9600
N2—C13	1.492 (10)	C12A—H12F	0.9600
N2—C13A	1.531 (5)	C13—H13A	0.9700
N2—H2	0.847 (10)	C13—H13B	0.9700
C1—H1B	0.9300	C13—C14	1.444 (16)
C1—C8	1.358 (4)	C14—H14A	0.9600
C2—C3	1.384 (4)	C14—H14B	0.9600
C2—C7	1.407 (3)	C14—H14C	0.9600
C3—H3	0.9300	C13A—H13C	0.9700
C3—C4	1.369 (4)	C13A—H13D	0.9700
C4—H4	0.9300	C13A—C14A	1.468 (6)
C4—C5	1.401 (4)	C14A—H14D	0.9600
C5—H5	0.9300	C14A—H14E	0.9600
C5—C6	1.367 (4)	C14A—H14F	0.9600
C6—C7	1.409 (3)	C15—C16	1.498 (3)
C7—C8	1.438 (4)	C16—H16	0.9300
C8—C9	1.499 (4)	C16—C17	1.301 (3)
C9—H9A	0.9700	C17—H17	0.9300
C9—H9B	0.9700	C17—C18	1.482 (3)
C9—C10	1.498 (4)		
			
C6—O1—H1	105 (3)	C12—C11—H11A	109.2
C18—O5—H5A	115 (3)	C12—C11—H11B	109.2
C1—N1—C2	109.0 (2)	C11—C12—H12A	109.5
C1—N1—H1A	131 (3)	C11—C12—H12B	109.5
C2—N1—H1A	119 (3)	C11—C12—H12C	109.5
C10—N2—C13A	107.4 (3)	H12A—C12—H12B	109.5
C10—N2—H2	107.6 (19)	H12A—C12—H12C	109.5
C11—N2—C10	111.4 (3)	H12B—C12—H12C	109.5
C11—N2—C13	94.9 (7)	N2—C11A—H11C	105.1
C11—N2—H2	109.0 (19)	N2—C11A—H11D	105.1
C11A—N2—C10	110.2 (12)	H11C—C11A—H11D	105.9
C11A—N2—C13A	129.0 (16)	C12A—C11A—N2	129 (3)
C11A—N2—H2	91 (3)	C12A—C11A—H11C	105.1
C13—N2—C10	117.6 (11)	C12A—C11A—H11D	105.1
C13—N2—H2	116 (2)	C11A—C12A—H12D	109.5
C13A—N2—H2	109.3 (19)	C11A—C12A—H12E	109.5
N1—C1—H1B	124.7	C11A—C12A—H12F	109.5
C8—C1—N1	110.7 (2)	H12D—C12A—H12E	109.5
C8—C1—H1B	124.7	H12D—C12A—H12F	109.5
N1—C2—C3	130.0 (2)	H12E—C12A—H12F	109.5
N1—C2—C7	107.1 (2)	N2—C13—H13A	108.4
C3—C2—C7	122.8 (3)	N2—C13—H13B	108.4
C2—C3—H3	121.4	H13A—C13—H13B	107.4
C4—C3—C2	117.3 (3)	C14—C13—N2	116 (3)
C4—C3—H3	121.4	C14—C13—H13A	108.4
C3—C4—H4	119.1	C14—C13—H13B	108.4
C3—C4—C5	121.8 (3)	C13—C14—H14A	109.5
C5—C4—H4	119.1	C13—C14—H14B	109.5
C4—C5—H5	119.7	C13—C14—H14C	109.5
C6—C5—C4	120.6 (3)	H14A—C14—H14B	109.5
C6—C5—H5	119.7	H14A—C14—H14C	109.5
O1—C6—C7	116.4 (2)	H14B—C14—H14C	109.5
C5—C6—O1	124.0 (2)	N2—C13A—H13C	108.7
C5—C6—C7	119.5 (2)	N2—C13A—H13D	108.7
C2—C7—C6	117.9 (2)	H13C—C13A—H13D	107.6
C2—C7—C8	107.3 (2)	C14A—C13A—N2	114.0 (4)
C6—C7—C8	134.7 (2)	C14A—C13A—H13C	108.7
C1—C8—C7	105.9 (2)	C14A—C13A—H13D	108.7
C1—C8—C9	125.6 (3)	C13A—C14A—H14D	109.5
C7—C8—C9	128.5 (2)	C13A—C14A—H14E	109.5
C8—C9—H9A	109.3	C13A—C14A—H14F	109.5
C8—C9—H9B	109.3	H14D—C14A—H14E	109.5
H9A—C9—H9B	108.0	H14D—C14A—H14F	109.5
C10—C9—C8	111.5 (2)	H14E—C14A—H14F	109.5
C10—C9—H9A	109.3	O2—C15—O3	123.7 (2)
C10—C9—H9B	109.3	O2—C15—C16	120.5 (2)
N2—C10—H10A	109.0	O3—C15—C16	115.8 (2)
N2—C10—H10B	109.0	C15—C16—H16	117.6
C9—C10—N2	112.8 (2)	C17—C16—C15	124.7 (2)
C9—C10—H10A	109.0	C17—C16—H16	117.6
C9—C10—H10B	109.0	C16—C17—H17	117.8
H10A—C10—H10B	107.8	C16—C17—C18	124.5 (2)
N2—C11—H11A	109.2	C18—C17—H17	117.8
N2—C11—H11B	109.2	O4—C18—O5	123.8 (2)
H11A—C11—H11B	107.9	O4—C18—C17	122.7 (2)
C12—C11—N2	111.9 (4)	O5—C18—C17	113.5 (2)
			
O1—C6—C7—C2	−175.9 (2)	C5—C6—C7—C8	178.3 (3)
O1—C6—C7—C8	1.1 (4)	C6—C7—C8—C1	−178.4 (3)
O2—C15—C16—C17	3.5 (4)	C6—C7—C8—C9	−0.4 (5)
O3—C15—C16—C17	−174.7 (3)	C7—C2—C3—C4	−0.1 (4)
N1—C1—C8—C7	0.2 (3)	C7—C8—C9—C10	76.0 (3)
N1—C1—C8—C9	−177.8 (2)	C8—C9—C10—N2	−177.2 (2)
N1—C2—C3—C4	179.4 (3)	C10—N2—C11—C12	−66.3 (5)
N1—C2—C7—C6	179.3 (2)	C10—N2—C11A—C12A	−52 (4)
N1—C2—C7—C8	1.5 (3)	C10—N2—C13—C14	169 (2)
C1—N1—C2—C3	179.1 (3)	C10—N2—C13A—C14A	172.6 (5)
C1—N1—C2—C7	−1.4 (3)	C11—N2—C10—C9	139.2 (4)
C1—C8—C9—C10	−106.4 (3)	C11—N2—C13—C14	−74 (2)
C2—N1—C1—C8	0.7 (3)	C11A—N2—C10—C9	117 (2)
C2—C3—C4—C5	1.2 (5)	C11A—N2—C13A—C14A	−51 (2)
C2—C7—C8—C1	−1.1 (3)	C13—N2—C10—C9	−112.9 (10)
C2—C7—C8—C9	176.9 (2)	C13—N2—C11—C12	171.5 (13)
C3—C2—C7—C6	−1.1 (4)	C13A—N2—C10—C9	−97.8 (4)
C3—C2—C7—C8	−178.9 (2)	C13A—N2—C11A—C12A	173 (3)
C3—C4—C5—C6	−1.1 (5)	C15—C16—C17—C18	176.1 (2)
C4—C5—C6—O1	176.7 (3)	C16—C17—C18—O4	−175.2 (3)
C4—C5—C6—C7	−0.2 (4)	C16—C17—C18—O5	3.9 (4)
C5—C6—C7—C2	1.2 (3)		

### Diethyl[2-(4-hydroxy-1H-indol-3-yl)ethyl]azanium 3-carboxyprop-2-enoate (4-HO-DET) Hydrogen-bond geometry (Å, º)

**Table:**

*D*—H···*A*	*D*—H	H···*A*	*D*···*A*	*D*—H···*A*
O5—H5*A*···O3^i^	0.90 (1)	1.70 (2)	2.559 (2)	157 (4)
O1—H1···O2	0.87 (1)	1.79 (2)	2.607 (3)	155 (4)
N1—H1*A*···O1^ii^	0.87 (1)	2.15 (1)	3.011 (3)	169 (3)
N2—H2···O3^iii^	0.85 (1)	1.96 (1)	2.783 (3)	165 (3)

Symmetry codes: (i) *x*, −*y*+3/2, *z*+1/2; (ii) *x*, −*y*+1/2, *z*+1/2; (iii) −*x*+1, −*y*+1, −*z*+1.

### Bis{[2-(4-acetoxy-1H-indol-3-yl)ethyl]diethylazanium} but-2-enedioate–(E)-butenedioic acid (1/1) (4-AcO-DET) Crystal data

**Table:**

2(C_16_H_23_N_2_O_2_^+^)·C_4_H_2_O_4_^2^^−^·C_4_H_4_O_4_	*F*(000) = 1664
*M**_r_* = 390.43	*D*_x_ = 1.256 Mg m^−^^3^
Monoclinic, *C*2/*c*	Mo *K*α radiation, λ = 0.71073 Å
*a* = 24.410 (2) Å	Cell parameters from 5098 reflections
*b* = 8.4508 (7) Å	θ = 2.6–25.3°
*c* = 21.445 (2) Å	µ = 0.09 mm^−^^1^
β = 110.963 (3)°	*T* = 300 K
*V* = 4131.1 (7) Å^3^	Block, colourless
*Z* = 8	0.18 × 0.15 × 0.12 mm

### Bis{[2-(4-acetoxy-1H-indol-3-yl)ethyl]diethylazanium} but-2-enedioate–(E)-butenedioic acid (1/1) (4-AcO-DET) Data collection

**Table:**

Bruker D8 Venture CMOS diffractometer	2420 reflections with *I* > 2σ(*I*)
φ and ω scans	*R*_int_ = 0.113
Absorption correction: multi-scan (SADABS; Krause *et al.*, 2015)	θ_max_ = 24.7°, θ_min_ = 2.6°
*T*_min_ = 0.697, *T*_max_ = 0.745	*h* = −28→28
47661 measured reflections	*k* = −9→9
3525 independent reflections	*l* = −25→25

### Bis{[2-(4-acetoxy-1H-indol-3-yl)ethyl]diethylazanium} but-2-enedioate–(E)-butenedioic acid (1/1) (4-AcO-DET) Refinement

**Table:**

Refinement on *F*^2^	Primary atom site location: structure-invariant direct methods
Least-squares matrix: full	Hydrogen site location: mixed
*R*[*F*^2^ > 2σ(*F*^2^)] = 0.048	H atoms treated by a mixture of independent and constrained refinement
*wR*(*F*^2^) = 0.122	*w* = 1/[σ^2^(*F*_o_^2^) + (0.0421*P*)^2^ + 4.0733*P*] where *P* = (*F*_o_^2^ + 2*F*_c_^2^)/3
*S* = 1.04	(Δ/σ)_max_ < 0.001
3525 reflections	Δρ_max_ = 0.33 e Å^−^^3^
268 parameters	Δρ_min_ = −0.17 e Å^−^^3^
9 restraints	

### Bis{[2-(4-acetoxy-1H-indol-3-yl)ethyl]diethylazanium} but-2-enedioate–(E)-butenedioic acid (1/1) (4-AcO-DET) Special details

**Table:**

Geometry. All esds (except the esd in the dihedral angle between two l.s. planes) are estimated using the full covariance matrix. The cell esds are taken into account individually in the estimation of esds in distances, angles and torsion angles; correlations between esds in cell parameters are only used when they are defined by crystal symmetry. An approximate (isotropic) treatment of cell esds is used for estimating esds involving l.s. planes.

### Bis{[2-(4-acetoxy-1H-indol-3-yl)ethyl]diethylazanium} but-2-enedioate–(E)-butenedioic acid (1/1) (4-AcO-DET) Fractional atomic coordinates and isotropic or equivalent isotropic displacement parameters (Å^2^)

**Table:**

	*x*	*y*	*z*	*U*_iso_*/*U*_eq_	
O1	0.74350 (9)	0.6039 (2)	0.61017 (9)	0.0633 (5)	
O2	0.76587 (11)	0.8498 (3)	0.59230 (16)	0.1071 (9)	
N1	0.85177 (9)	0.5727 (3)	0.83707 (11)	0.0508 (5)	
H1	0.8769 (10)	0.528 (3)	0.8721 (9)	0.072 (9)*	
N2	0.59712 (8)	0.5647 (2)	0.67889 (10)	0.0430 (5)	
H2	0.5887 (11)	0.651 (2)	0.6524 (10)	0.057 (8)*	
C1	0.79489 (10)	0.6118 (3)	0.82720 (12)	0.0477 (6)	
H1A	0.779946	0.624586	0.861145	0.057*	
C2	0.85802 (10)	0.5604 (3)	0.77654 (13)	0.0444 (6)	
C3	0.90724 (12)	0.5215 (3)	0.76154 (16)	0.0589 (7)	
H3	0.943156	0.500534	0.795016	0.071*	
C4	0.90072 (15)	0.5153 (3)	0.69572 (19)	0.0721 (9)	
H4	0.932990	0.490469	0.684192	0.087*	
C5	0.84718 (15)	0.5451 (3)	0.64554 (16)	0.0678 (8)	
H5	0.843846	0.537899	0.601061	0.081*	
C6	0.79898 (12)	0.5854 (3)	0.66107 (13)	0.0515 (7)	
C7	0.80299 (10)	0.5954 (2)	0.72714 (12)	0.0406 (6)	
C8	0.76320 (10)	0.6296 (3)	0.76112 (11)	0.0398 (5)	
C9	0.69942 (10)	0.6730 (3)	0.73296 (13)	0.0464 (6)	
H9A	0.693496	0.755282	0.699628	0.056*	
H9B	0.687531	0.714640	0.768327	0.056*	
C10	0.66171 (9)	0.5311 (3)	0.70143 (13)	0.0449 (6)	
H10A	0.671155	0.496520	0.663330	0.054*	
H10B	0.671103	0.445124	0.733515	0.054*	
C11	0.57684 (11)	0.6015 (3)	0.73551 (13)	0.0527 (7)	
H11A	0.535407	0.627464	0.717297	0.063*	
H11B	0.597624	0.694451	0.758699	0.063*	
C12	0.58588 (15)	0.4689 (4)	0.78553 (16)	0.0786 (9)	
H12A	0.571742	0.501205	0.819968	0.118*	
H12B	0.626920	0.444336	0.804997	0.118*	
H12C	0.564717	0.376855	0.763378	0.118*	
C13	0.56146 (12)	0.4341 (3)	0.63634 (13)	0.0574 (7)	
H13A	0.572445	0.334770	0.660275	0.069*	
H13B	0.520334	0.452764	0.628564	0.069*	
C14	0.56919 (15)	0.4199 (4)	0.57135 (16)	0.0833 (10)	
H14A	0.541126	0.345901	0.543643	0.125*	
H14B	0.608145	0.383101	0.578315	0.125*	
H14C	0.563306	0.521349	0.549841	0.125*	
C15	0.73154 (15)	0.7429 (4)	0.57790 (16)	0.0655 (8)	
C16	0.67192 (15)	0.7417 (4)	0.52599 (16)	0.0836 (10)	
H16A	0.669563	0.660156	0.493983	0.125*	
H16B	0.664099	0.842500	0.503877	0.125*	
H16C	0.643501	0.721812	0.546504	0.125*	
O3	0.48170 (7)	0.7667 (2)	0.59379 (9)	0.0618 (5)	
O4	0.56861 (6)	0.82722 (19)	0.59214 (8)	0.0473 (4)	
O5	0.37498 (8)	0.6824 (2)	0.56621 (10)	0.0696 (6)	
H5A	0.4125 (8)	0.724 (4)	0.5712 (17)	0.109 (12)*	
O6	0.34179 (7)	0.8613 (2)	0.48661 (9)	0.0620 (5)	
C17	0.51391 (9)	0.8438 (3)	0.57089 (11)	0.0376 (5)	
C18	0.48603 (9)	0.9610 (2)	0.51631 (11)	0.0387 (5)	
H18	0.446073	0.979934	0.504643	0.046*	
C19	0.33287 (10)	0.7592 (3)	0.52073 (12)	0.0418 (6)	
C20	0.27361 (9)	0.7103 (3)	0.51714 (12)	0.0450 (6)	
H20	0.269777	0.619973	0.540034	0.054*	

### Bis{[2-(4-acetoxy-1H-indol-3-yl)ethyl]diethylazanium} but-2-enedioate–(E)-butenedioic acid (1/1) (4-AcO-DET) Atomic displacement parameters (Å^2^)

**Table:**

	*U*^11^	*U*^22^	*U*^33^	*U*^12^	*U*^13^	*U*^23^
O1	0.0847 (14)	0.0524 (11)	0.0450 (10)	−0.0030 (10)	0.0135 (10)	0.0114 (9)
O2	0.0861 (17)	0.0642 (15)	0.176 (3)	0.0127 (13)	0.0530 (18)	0.0485 (16)
N1	0.0401 (13)	0.0544 (13)	0.0471 (14)	−0.0021 (10)	0.0024 (10)	0.0022 (11)
N2	0.0359 (11)	0.0347 (11)	0.0512 (12)	0.0040 (9)	0.0070 (9)	0.0036 (10)
C1	0.0422 (15)	0.0537 (15)	0.0448 (15)	−0.0044 (11)	0.0124 (12)	−0.0024 (12)
C2	0.0412 (14)	0.0329 (12)	0.0565 (16)	−0.0031 (10)	0.0142 (12)	0.0018 (11)
C3	0.0479 (16)	0.0450 (15)	0.085 (2)	0.0015 (12)	0.0248 (15)	0.0063 (14)
C4	0.072 (2)	0.0570 (18)	0.106 (3)	0.0061 (15)	0.054 (2)	0.0070 (18)
C5	0.095 (3)	0.0536 (17)	0.070 (2)	0.0008 (16)	0.049 (2)	0.0010 (15)
C6	0.0646 (18)	0.0391 (14)	0.0497 (16)	−0.0002 (12)	0.0193 (14)	0.0042 (11)
C7	0.0439 (14)	0.0281 (11)	0.0475 (14)	−0.0028 (10)	0.0137 (11)	0.0028 (10)
C8	0.0381 (13)	0.0338 (12)	0.0426 (14)	−0.0017 (10)	0.0085 (11)	−0.0007 (10)
C9	0.0413 (14)	0.0396 (13)	0.0533 (15)	0.0046 (11)	0.0108 (12)	−0.0008 (11)
C10	0.0340 (13)	0.0383 (13)	0.0566 (15)	0.0076 (10)	0.0092 (11)	0.0005 (11)
C11	0.0453 (15)	0.0516 (15)	0.0619 (17)	0.0053 (12)	0.0199 (13)	−0.0004 (13)
C12	0.091 (2)	0.080 (2)	0.069 (2)	0.0118 (18)	0.0329 (18)	0.0145 (17)
C13	0.0501 (16)	0.0460 (14)	0.0623 (18)	−0.0023 (12)	0.0032 (13)	−0.0032 (13)
C14	0.088 (2)	0.071 (2)	0.070 (2)	0.0002 (18)	0.0037 (18)	−0.0170 (17)
C15	0.079 (2)	0.0580 (19)	0.076 (2)	0.0190 (17)	0.0485 (18)	0.0212 (16)
C16	0.092 (3)	0.097 (2)	0.070 (2)	0.030 (2)	0.0385 (19)	0.0336 (19)
O3	0.0325 (9)	0.0784 (13)	0.0661 (12)	−0.0012 (9)	0.0076 (9)	0.0319 (10)
O4	0.0281 (9)	0.0491 (10)	0.0560 (10)	0.0027 (7)	0.0043 (8)	0.0108 (8)
O5	0.0346 (10)	0.0803 (14)	0.0847 (14)	0.0017 (10)	0.0101 (10)	0.0404 (11)
O6	0.0397 (10)	0.0781 (13)	0.0660 (12)	−0.0010 (9)	0.0162 (9)	0.0282 (10)
C17	0.0280 (13)	0.0395 (12)	0.0386 (13)	−0.0021 (10)	0.0036 (10)	−0.0011 (10)
C18	0.0254 (12)	0.0387 (13)	0.0445 (14)	0.0012 (9)	0.0033 (9)	0.0008 (10)
C19	0.0341 (13)	0.0468 (14)	0.0425 (14)	0.0025 (11)	0.0110 (11)	0.0052 (12)
C20	0.0362 (13)	0.0505 (15)	0.0482 (15)	−0.0020 (10)	0.0148 (11)	0.0068 (11)

### Bis{[2-(4-acetoxy-1H-indol-3-yl)ethyl]diethylazanium} but-2-enedioate–(E)-butenedioic acid (1/1) (4-AcO-DET) Geometric parameters (Å, º)

**Table:**

O1—C6	1.412 (3)	C11—H11A	0.9700
O1—C15	1.342 (3)	C11—H11B	0.9700
O2—C15	1.195 (4)	C11—C12	1.512 (4)
N1—H1	0.868 (10)	C12—H12A	0.9600
N1—C1	1.368 (3)	C12—H12B	0.9600
N1—C2	1.364 (3)	C12—H12C	0.9600
N2—H2	0.906 (10)	C13—H13A	0.9700
N2—C10	1.502 (3)	C13—H13B	0.9700
N2—C11	1.499 (3)	C13—C14	1.477 (4)
N2—C13	1.497 (3)	C14—H14A	0.9600
C1—H1A	0.9300	C14—H14B	0.9600
C1—C8	1.358 (3)	C14—H14C	0.9600
C2—C3	1.390 (4)	C15—C16	1.483 (4)
C2—C7	1.413 (3)	C16—H16A	0.9600
C3—H3	0.9300	C16—H16B	0.9600
C3—C4	1.364 (4)	C16—H16C	0.9600
C4—H4	0.9300	O3—C17	1.249 (3)
C4—C5	1.387 (4)	O4—C17	1.255 (3)
C5—H5	0.9300	O5—H5A	0.952 (10)
C5—C6	1.375 (4)	O5—C19	1.308 (3)
C6—C7	1.387 (3)	O6—C19	1.201 (3)
C7—C8	1.437 (3)	C17—C18	1.498 (3)
C8—C9	1.500 (3)	C18—C18^i^	1.316 (4)
C9—H9A	0.9700	C18—H18	0.9300
C9—H9B	0.9700	C19—C20	1.480 (3)
C9—C10	1.516 (3)	C20—C20^ii^	1.309 (4)
C10—H10A	0.9700	C20—H20	0.9300
C10—H10B	0.9700		
			
C15—O1—C6	117.9 (2)	N2—C11—H11A	108.6
C1—N1—H1	127 (2)	N2—C11—H11B	108.6
C2—N1—H1	120 (2)	N2—C11—C12	114.5 (2)
C2—N1—C1	108.9 (2)	H11A—C11—H11B	107.6
C10—N2—H2	109.3 (16)	C12—C11—H11A	108.6
C11—N2—H2	106.0 (16)	C12—C11—H11B	108.6
C11—N2—C10	113.05 (19)	C11—C12—H12A	109.5
C13—N2—H2	105.2 (16)	C11—C12—H12B	109.5
C13—N2—C10	111.80 (18)	C11—C12—H12C	109.5
C13—N2—C11	111.0 (2)	H12A—C12—H12B	109.5
N1—C1—H1A	124.6	H12A—C12—H12C	109.5
C8—C1—N1	110.9 (2)	H12B—C12—H12C	109.5
C8—C1—H1A	124.6	N2—C13—H13A	109.0
N1—C2—C3	129.7 (2)	N2—C13—H13B	109.0
N1—C2—C7	107.3 (2)	H13A—C13—H13B	107.8
C3—C2—C7	123.0 (3)	C14—C13—N2	113.1 (2)
C2—C3—H3	121.4	C14—C13—H13A	109.0
C4—C3—C2	117.3 (3)	C14—C13—H13B	109.0
C4—C3—H3	121.4	C13—C14—H14A	109.5
C3—C4—H4	119.2	C13—C14—H14B	109.5
C3—C4—C5	121.7 (3)	C13—C14—H14C	109.5
C5—C4—H4	119.2	H14A—C14—H14B	109.5
C4—C5—H5	119.8	H14A—C14—H14C	109.5
C6—C5—C4	120.5 (3)	H14B—C14—H14C	109.5
C6—C5—H5	119.8	O1—C15—C16	110.8 (3)
C5—C6—O1	120.5 (3)	O2—C15—O1	121.7 (3)
C5—C6—C7	120.6 (3)	O2—C15—C16	127.5 (3)
C7—C6—O1	118.7 (2)	C15—C16—H16A	109.5
C2—C7—C8	107.2 (2)	C15—C16—H16B	109.5
C6—C7—C2	117.0 (2)	C15—C16—H16C	109.5
C6—C7—C8	135.8 (2)	H16A—C16—H16B	109.5
C1—C8—C7	105.7 (2)	H16A—C16—H16C	109.5
C1—C8—C9	124.7 (2)	H16B—C16—H16C	109.5
C7—C8—C9	129.6 (2)	C19—O5—H5A	111 (2)
C8—C9—H9A	109.4	O3—C17—O4	122.5 (2)
C8—C9—H9B	109.4	O3—C17—C18	118.56 (19)
C8—C9—C10	111.29 (19)	O4—C17—C18	119.0 (2)
H9A—C9—H9B	108.0	C17—C18—H18	117.6
C10—C9—H9A	109.4	C18^i^—C18—C17	124.7 (3)
C10—C9—H9B	109.4	C18^i^—C18—H18	117.6
N2—C10—C9	113.21 (18)	O5—C19—C20	113.1 (2)
N2—C10—H10A	108.9	O6—C19—O5	123.1 (2)
N2—C10—H10B	108.9	O6—C19—C20	123.8 (2)
C9—C10—H10A	108.9	C19—C20—H20	118.9
C9—C10—H10B	108.9	C20^ii^—C20—C19	122.3 (3)
H10A—C10—H10B	107.7	C20^ii^—C20—H20	118.9
			
O1—C6—C7—C2	173.7 (2)	C5—C6—C7—C8	−178.5 (3)
O1—C6—C7—C8	−3.8 (4)	C6—O1—C15—O2	−1.6 (4)
N1—C1—C8—C7	1.4 (3)	C6—O1—C15—C16	179.6 (2)
N1—C1—C8—C9	−179.8 (2)	C6—C7—C8—C1	176.9 (3)
N1—C2—C3—C4	178.9 (3)	C6—C7—C8—C9	−1.9 (4)
N1—C2—C7—C6	−178.2 (2)	C7—C2—C3—C4	−1.0 (4)
N1—C2—C7—C8	0.0 (2)	C7—C8—C9—C10	73.5 (3)
C1—N1—C2—C3	−179.1 (2)	C8—C9—C10—N2	174.0 (2)
C1—N1—C2—C7	0.8 (3)	C10—N2—C11—C12	−61.4 (3)
C1—C8—C9—C10	−105.1 (3)	C10—N2—C13—C14	−67.2 (3)
C2—N1—C1—C8	−1.4 (3)	C11—N2—C10—C9	−65.1 (3)
C2—C3—C4—C5	−0.5 (4)	C11—N2—C13—C14	165.6 (2)
C2—C7—C8—C1	−0.8 (2)	C13—N2—C10—C9	168.8 (2)
C2—C7—C8—C9	−179.6 (2)	C13—N2—C11—C12	65.1 (3)
C3—C2—C7—C6	1.8 (3)	C15—O1—C6—C5	−82.6 (3)
C3—C2—C7—C8	180.0 (2)	C15—O1—C6—C7	102.8 (3)
C3—C4—C5—C6	1.3 (4)	O3—C17—C18—C18^i^	−173.5 (3)
C4—C5—C6—O1	−175.1 (2)	O4—C17—C18—C18^i^	6.9 (4)
C4—C5—C6—C7	−0.6 (4)	O5—C19—C20—C20^ii^	168.7 (3)
C5—C6—C7—C2	−0.9 (3)	O6—C19—C20—C20^ii^	−9.7 (5)

Symmetry codes: (i) −*x*+1, −*y*+2, −*z*+1; (ii) −*x*+1/2, −*y*+3/2, −*z*+1.

### Bis{[2-(4-acetoxy-1H-indol-3-yl)ethyl]diethylazanium} but-2-enedioate–(E)-butenedioic acid (1/1) (4-AcO-DET) Hydrogen-bond geometry (Å, º)

**Table:**

*D*—H···*A*	*D*—H	H···*A*	*D*···*A*	*D*—H···*A*
N1—H1···O4^iii^	0.87 (1)	2.12 (2)	2.876 (3)	145 (3)
N2—H2···O3	0.91 (1)	2.65 (2)	3.241 (2)	124 (2)
N2—H2···O4	0.91 (1)	1.91 (1)	2.818 (2)	177 (3)
O5—H5*A*···O3	0.95 (1)	1.62 (1)	2.558 (2)	167 (3)

Symmetry code: (iii) −*x*+3/2, *y*−1/2, −*z*+3/2.
